# Chance-Type Fracture of the Sacrum in an Elderly Patient: A Case Report

**DOI:** 10.7759/cureus.98344

**Published:** 2025-12-02

**Authors:** Yasuhito Asaka, Ryota Kimura, Yuji Kasukawa, Michio Hongo, Naohisa Miyakoshi

**Affiliations:** 1 Orthopedic Surgery, Akita University Graduate School of Medicine, Akita, JPN; 2 Rehabilitation Medicine, Akita University Graduate School of Medicine, Akita, JPN; 3 Physical Therapy, Akita University Graduate School of Medicine, Akita, JPN

**Keywords:** chance fracture, flexion-distraction injury, osteoporosis, posterior ligamentous complex injuries, sacral fracture, sacrum

## Abstract

Chance-type fractures involving the sacrum have not been previously reported to the best of our knowledge. We describe a rare case in a 79-year-old man who fell into deep snow, the pelvis became effectively fixed, and the trunk flexed forward, creating a seat-belt-like flexion-distraction mechanism, resulting in low back and left leg pain. Initial plain radiographs were normal, and the patient was managed conservatively for a presumed sacral fracture. Progressive bilateral buttock and leg pain prompted magnetic resonance imaging (MRI) at four weeks, which demonstrated marrow edema in S1, fracture of the L5 spinous process, and posterior ligamentous complex (PLC) disruption, although the PLC injury was initially unrecognized. Persistent pain led to computed tomography (CT) at 18 weeks, revealing a horizontal fracture across L5-S1 with nonunion, clarifying the diagnosis and demonstrating mechanical instability. Because nonunion and PLC rupture predicted failure of further conservative care, we performed posterior fixation from L4 to S2 combined with vertebroplasty of S1. The procedure immediately relieved pain; CT confirmed solid union at one year, and the patient regained independent ambulation and full activities of daily living at two years. Although the sacrum comprises fused sacral vertebrae, flexion forces can concentrate at S1 owing to L5-S1 mobility, permitting Chance-type injury. Such fractures are likely underdiagnosed when assessment relies only on plain radiographs. This diagnostic sequence-normal radiographs, abnormal MRI, and confirmatory CT, highlights the importance of early cross-sectional imaging. In elderly patients with suspected sacral fractures, early MRI to assess PLC integrity and timely stabilization when instability is present should be considered.

## Introduction

The annual incidence of traumatic sacral fractures in the United States rose from 0.67 per 100,000 population in 2002 to 2.09 per 100,000 in 2011, representing nearly a threefold increase over a decade [1]. These fractures are frequently missed or diagnosed late, with reported rates ranging from 25% to 70%, because of non-specific symptoms and the limited sensitivity of plain radiographs [2]. Flexion-distraction injuries occur when the anterior spinal column fails under compression while the posterior elements fail under tension, producing a characteristic horizontal fracture line. In 1948, Chance described a flexion-distraction injury that was purely osseous, now termed the Chance fracture, characterized by a horizontal fracture line extending through the posterior elements into the vertebral body [3]. This classic injury pattern is now distinguished from broader seat-belt-type (flexion-distraction) injuries that can involve osseoligamentous failure across one or two motion segments (Chance-type fracture) [4]. Within Denis’ three-column framework, flexion-distraction injuries are categorized as seat-belt-type, in which failure of the middle/posterior columns indicates mechanical instability [5]. Such injuries typically result from sudden forward-flexion forces, most commonly during motor vehicle collisions in which a seat belt serves as the fulcrum [6], and they usually occur at the thoracolumbar junction [7]. A Chance-type fracture involving the sacrum appears to be exceedingly rare and, to the best of our knowledge, has not been previously reported. We describe a case of sacral flexion-distraction (Chance-type fracture) that progressed to nonunion.

## Case presentation

**Figure 1:**
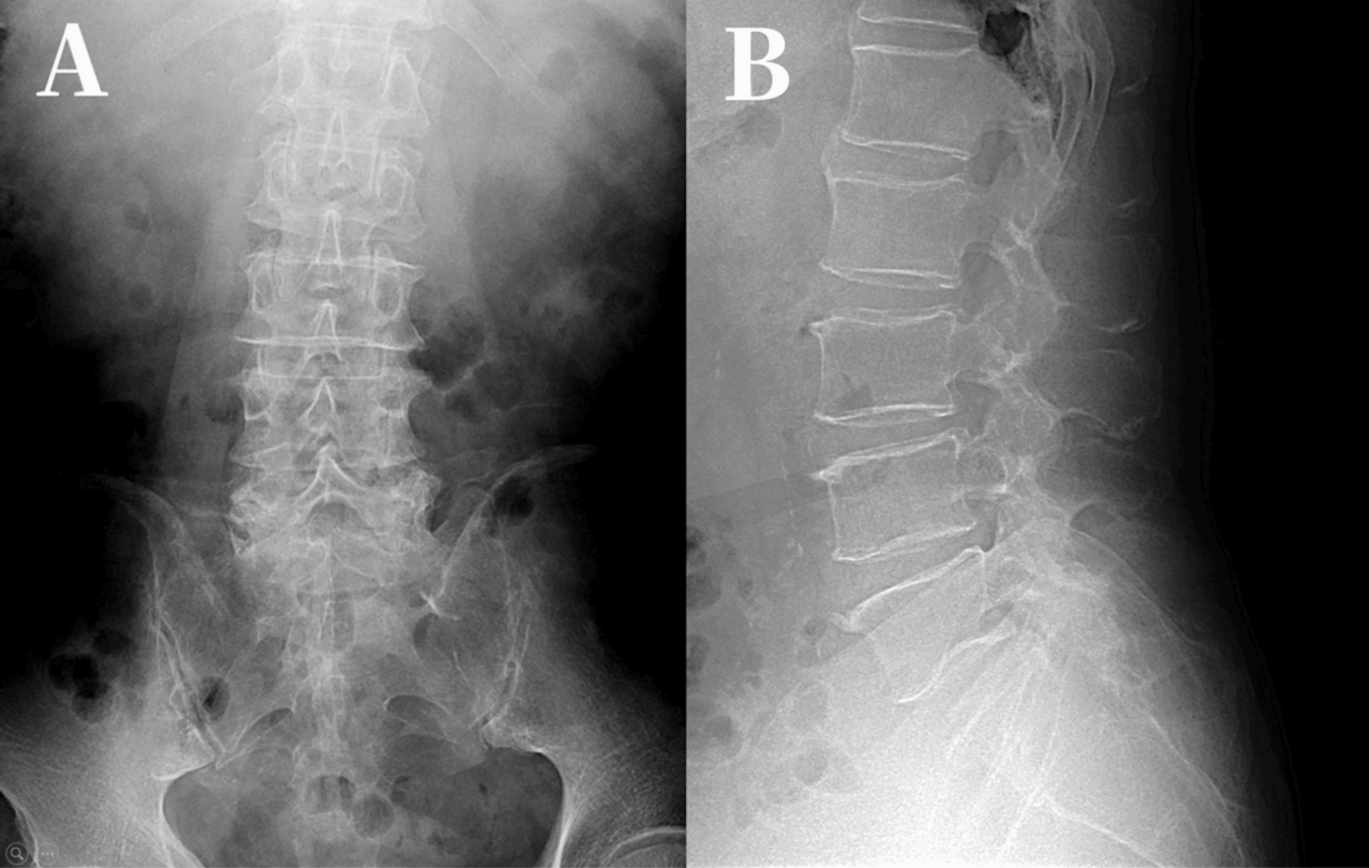
Anteroposterior and Lateral images. (A) Anteroposterior (B) Lateral radiographs showing no apparent fracture (10 days after injury).

A 79-year-old man fell into deep snow. The pelvis became effectively fixed while the trunk flexed forward, creating a seat-belt-like flexion-distraction mechanism. He developed pain in the left buttock and lateral thigh and presented to a local clinic 10 days later. A sacral fracture was diagnosed (Figure 1), and conservative treatment was initiated.

**Figure 2:**
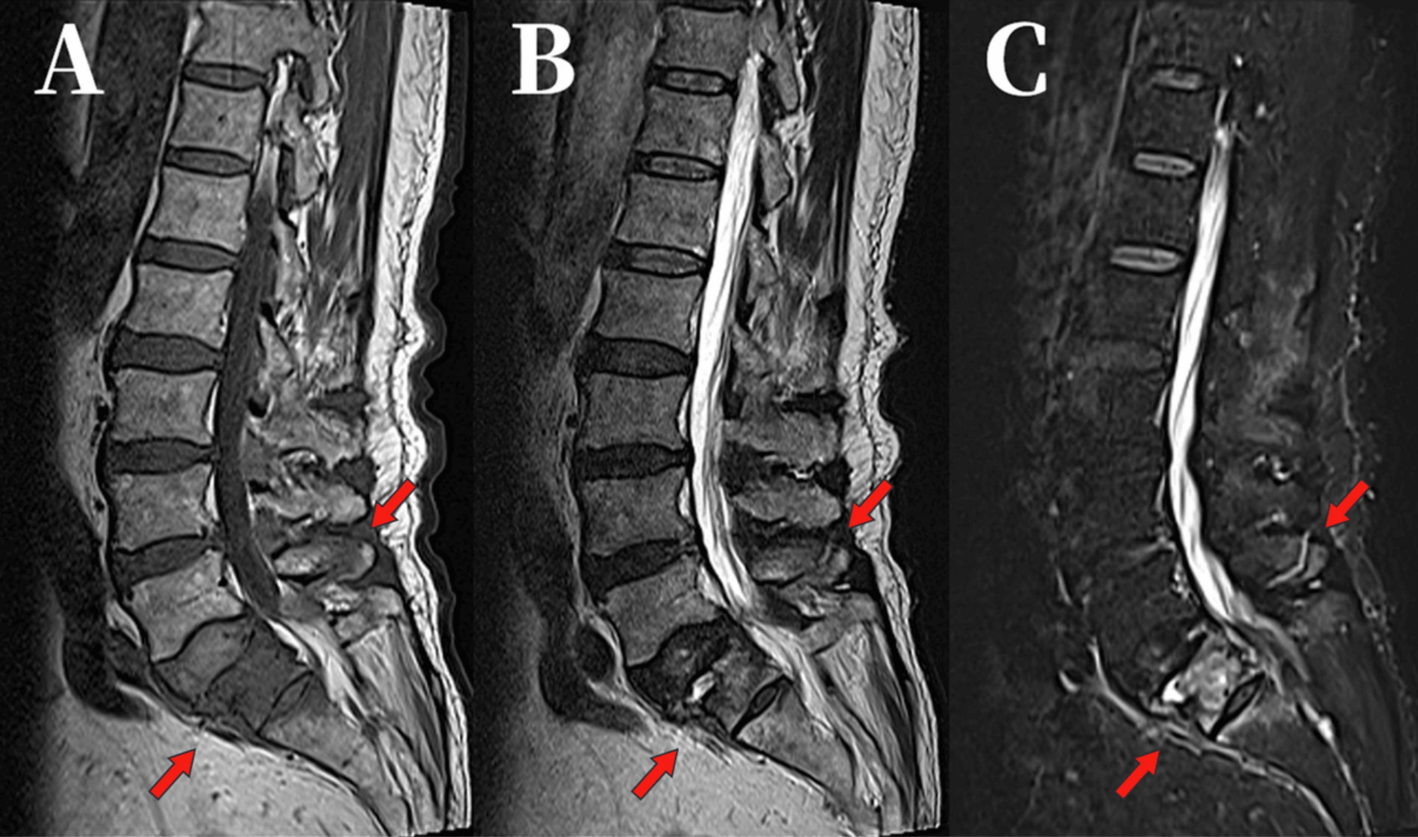
Sagittal MRI images. (A) T1-weighted (B) T2-weighted (C) STIR images showing diffuse marrow edema in S1 and an L5 spinous process fracture (4 weeks after injury). MRI: magnetic resonance imaging, STIR: short tau inversion recovery.

However, his pain gradually extended to the right buttock, thigh, and lower leg, leading to difficulty with ambulation. He was admitted to the same hospital six weeks after the injury. MRI demonstrated extensive signal changes within the S1 vertebral body, an L5 spinous process fracture, and disruption of the posterior ligamentous complex (PLC), as shown in Figure 2, although the latter was initially overlooked.

**Figure 3:**
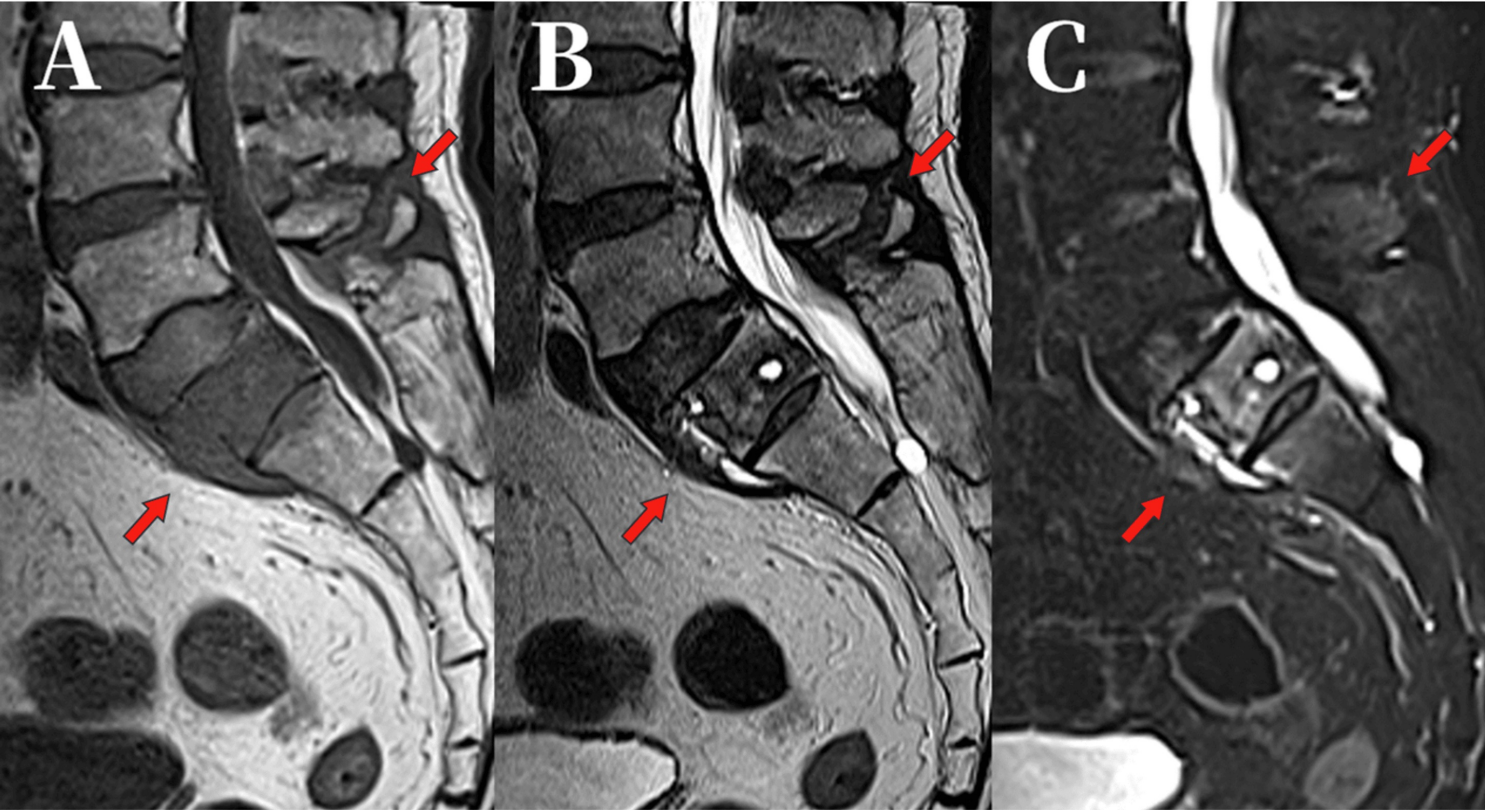
Sagittal MRI images. (A) T1-weighted (B) T2-weighted (C) STIR showing persistent signal changes within the vertebral body without improvement (12 weeks after injury). MRI: magnetic resonance imaging, STIR: short tau inversion recovery.

Despite symptomatic improvement with conservative therapy, MRI at 12 weeks showed no interval healing and raised concern for a bone tumor (Figure 3), prompting referral to our hospital. The MRI appearance was not typical of a simple osteoporotic fracture, and progressive expansion of the signal abnormality over time further raised concern for an underlying pathological process, including malignancy.

**Figure 4:**
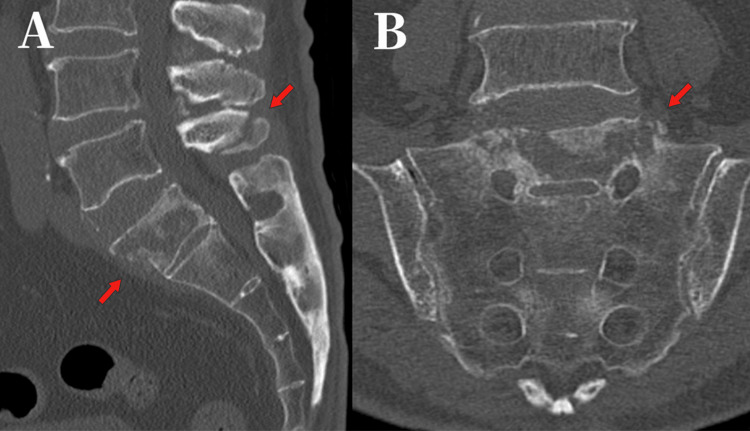
Sagittal and Coronal images. (A) Sagittal (B) Coronal views of the fracture site showing nonunion (18 weeks after injury). CT: computed tomography.

Computed tomography (CT) at 18 weeks demonstrated progression to nonunion (Figure 4). We chose surgical intervention because the fracture showed significant instability involving the PLC and had progressed to nonunion, resulting in persistent pain.

**Figure 5:**
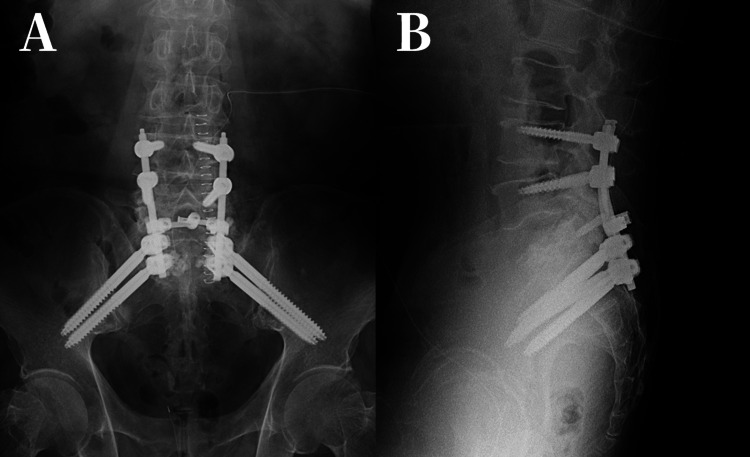
Anteroposterior and Lateral images. (A) Anteroposterior (B) Lateral radiographs (the day of surgery).

We performed posterior fixation from L4 to S2 with S1 vertebroplasty (Figure 5). Intraoperative biopsy of the fracture site showed no evidence of malignancy. 

**Figure 6:**
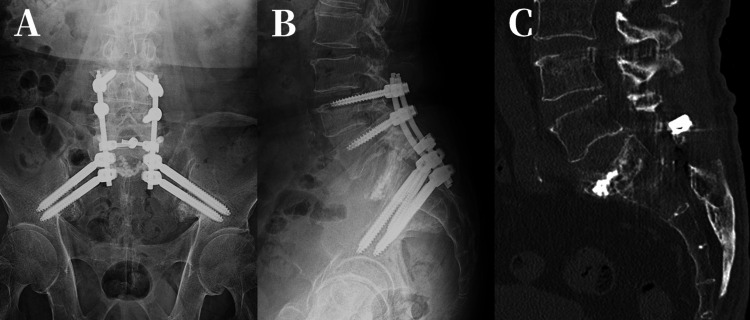
Anteroposterior, Lateral and Sagittal images. (A) Anteroposterior (B) Lateral (C) Sagittal CT image showing bone union (1 year after surgery). CT: computed tomography.

Postoperatively, symptoms improved rapidly. CT at one year confirmed bone union (Figure 6), and by two years, the patient was able to walk independently and perform full activities of daily living.

## Discussion

Chance fractures result from sudden flexion-distraction forces that generate a horizontal fracture line extending from the posterior elements (spinous process and ligaments) through the vertebral body and disc. This pattern arises because the intervertebral discs and facet joints permit anteroposterior motion, concentrating mechanical stress at the injured segment. The sacrum, formed by fused sacral vertebrae and stabilized by sacroiliac joints and multiple ligaments, has limited mobility and is generally protected from flexion stress. However, because L5-S1 retains mobility through its disc and facet joints, flexion forces can focus on the S1 vertebral body, theoretically allowing a Chance-type fracture to occur [3].

To our knowledge, sacral Chance-type fractures have not been previously reported, but anatomical considerations support their plausibility. In our case, a continuous fracture involving ligamentous elements extended from the L5 spinous process to the S1 body, leading to the diagnosis of this rare sacral Chance-type fracture. Typical Chance fractures of the thoracolumbar spine are usually caused by high-energy trauma, such as motor vehicle collisions or falls from height, most often in younger patients, although occasional cases in older adults after low-energy trauma have been described [8]. Chance fractures are classified as osseous, ligamentous, or mixed types [6]. Surgical treatment is generally indicated when instability or neurological deficits are present. The Thoracolumbar Injury Classification and Severity Score assigns points based on fracture morphology, PLC integrity, and neurological status; a score of 5 or higher indicates the need for surgical treatment [9].

In our case, the fracture scored 9 points (morphology 4, PLC 3, neurological 2), clearly meeting surgical criteria. However, because the PLC injury was not initially recognized, the fracture was managed conservatively and progressed to nonunion. Even fractures that appear osteoporotic on initial imaging may involve posterior element disruption, as demonstrated in this case. Early MRI evaluation is therefore critical to assess PLC integrity and determine the need for timely surgical stabilization. Posterior multilevel fixation is the standard surgical approach. When sacral involvement compromises lumbosacral stability, S2-alar-iliac anchorage can be added to enhance distal fixation. Although Kümmell disease, which involves delayed vertebral collapse with intravertebral cleft formation, may be considered in elderly patients, it was unlikely in our case because no intravertebral vacuum cleft or other characteristic findings were present.

## Conclusions

Chance-type fractures can occur in the sacrum when flexion forces are applied to the lumbar spine while the pelvis is fixed. This case demonstrates that such a mechanism can produce a flexion-distraction injury involving the sacrum. In elderly patients with vertebral fractures, MRI should be routinely performed to assess the integrity of the PLC, and early surgical fixation should be considered when instability is identified.

## References

[1] Bydon M, De la Garza-Ramos R, Macki M, Desai A, Gokaslan AK, Bydon A (2014). Incidence of sacral fractures and in-hospital postoperative complications in the United States: an analysis of 2002-2011 data. Spine (Phila Pa 1976).

[2] Santolini E, Kanakaris NK, Giannoudis PV (2020). Sacral fractures: issues, challenges, solutions. EFORT Open Rev.

[3] CHance GQ (1948). Note on a type of flexion fracture of the spine. Br J Radiol.

[4] GA JW, BR PW (1962). The seat belt syndrome. J Trauma.

[5] Denis F (1983). The three column spine and its significance in the classification of acute thoracolumbar spinal injuries. Spine (Phila Pa 1976).

[6] Bernstein MP, Mirvis SE, Shanmuganathan K (2006). Chance-type fractures of the thoracolumbar spine: imaging analysis in 53 patients. AJR Am J Roentgenol.

[7] Chapman JR, Agel J, Jurkovich GJ, Bellabarba C (2008). Thoracolumbar flexion-distraction injuries: associated morbidity and neurological outcomes. Spine (Phila Pa 1976).

[8] Zwolak P, Kröber M (2016). Chance fracture in an older patient with positive sagittal imbalance and previous lumbar arthrodesis: what can be done?. Arch Orthop Trauma Surg.

[9] Vaccaro AR, Lehman RA Jr, Hurlbert RJ (2005). A new classification of thoracolumbar injuries: the importance of injury morphology, the integrity of the posterior ligamentous complex, and neurologic status. Spine (Phila Pa 1976).

